# Alteration of Methanogenic Archaeon by Ethanol Contribute to the Enhancement of Biogenic Methane Production of Lignite

**DOI:** 10.3389/fmicb.2019.02323

**Published:** 2019-10-10

**Authors:** Xiuqing Yang, Qi Liang, Yanmei Chen, Baoyu Wang

**Affiliations:** ^1^Key Laboratory of Chemical Biology and Molecular Engineering of the Ministry of Education, Institute of Biotechnology, Shanxi University, Taiyuan, China; ^2^State Key Laboratory of Coal and CBM Co-Mining, Jincheng, China; ^3^Yi’an Lanyan Coal and Coalbed Methane Simultaneous Extraction Technology Co., Ltd., Jincheng, China

**Keywords:** coalbed methane (CBM), ethanol, enhanced biogenic methane, microbial community, physicochemical property

## Abstract

Bioconverting coal to methane is a green and environmental friendly method to reuse waste coal. In this study, heterologous bacteria were used for the gas-producing fermentation of lignite under laboratory conditions, simultaneously, different concentrations of ethanol added into the culture to investigate the effect of ethanol on gas production and microbial flora structure. Results show that when the ethanol concentration was 1%, the best methanogenesis was achieved at 44.86 mL/g, which was twice the gas production of 0% ethanol. Before and after gas fermentation, the composition and structure of the coal changed, the volatile matter and fixed carbon increased, and the ash decreased. The absorbance value at characteristic peaks of all functional groups decreased, new peaks were generated at 2,300/cm, and the peak value disappeared at 3,375/cm. Thus, microorganisms interacted with coal, consumed it, and produced new materials. The microbial flora changes during gas production were tracked in real time. 0.5 and 1% ethanol did not obviously change the bacterial communities but strongly influenced the archaeon communities, thereby changed the methane production pathway. In the absence of ethanol, *Methanosarcina* was continuously increasing with the extension of fermentation time, this pathway was the nutrient type of acetic acid. When ethanol was added, *Methanobacterium* gradually increased, the pathway was mainly hydrotropic type. In summary, adding ethanol can increase the coalbed methane production, change the structure and composition of coal, and facilitate the interaction of microbe with coal. Therefore, the methanogenic archaeon changes could help improve the methane-producing ability of lignite in the presence of ethanol.

## Introduction

Coalbed methane (CBM) is an unconventional and self-preserving natural gas with methane as its main component. This gas has received considerable attention as a new type of clean energy. China’s CBM reserves are next to Russia and Canada, ranking third worldwide. Although abundant, CBM has low extraction and utilization rates due to the long-term unreasonable mining in the early stage. These unfavorable factors limit the development of the CBM industry.

To fully utilize CBM resources, researchers have developed considerably numerous CBM technologies. The microbial stimulation of CBM technology has emerged among many because of its environmental protection and pollution free characteristics. Many methods can be used to increase CBM by using microorganisms. Adding nutrients such as main minerals, trace metals, and vitamins to the coal seam stimulates the microbial flora to metabolize coal for methane production (Ünal et al., 2012; Fallgren et al., 2013; Zhang et al., 2016b). Microbial activity can also be increased by adding new or additional microorganisms to enhance CBM production (Jones et al., 2008). In addition, microorganisms can further interact with coal by changing the fermentation environment, such as changing pH, temperature, and particle size of coal (Green et al., 2008; Gupta and Gupta, 2014).

As a non-toxic and inexpensive organic material, ethanol has attracted the attention of researchers. Bi et al. (2017) enhanced microbial activity by finding the best nutrient-based formula. The authors added surfactants (Tween-20 and SDS), organic solvents (ethanol, methanol, and isopropanol), and carbon sources (sodium formate and sodium acetate) to the culture medium to stimulate microbial activity; consequently, the three alcohols and sodium acetate were proven to be essential for maximizing the production of methane from coal (Bi et al., 2017). Ethanol has a statistically significant dose-dependent effect in increasing methane production. At 100 mM, ethanol increases methane yield by at least 24 times, but no further change was noted at 300 mM concentration (Zhang et al., 2016a). Similarly, Liu Y et al. discovered that when 5 or 10 mg of ethanol was added to 10 g of coal from Powder River Basin, the production of methane increased (Liu et al., 2013).

Since (Shimizu et al., 2007) first described the microbial community associated with CBM in Northern Japan, many studies on the microbial community of CBM have been reported. Ünal et al. (2012) found that the proper addition of trace elements could promote CBM production; the main methanogenic bacteria were *Methanobacterium subterraneum* and *Methanobacterium formicicum* after cultivation. Exogenous microorganisms could increase the production of CBM, and methane production correlates with the growth of *Methanosaeta concilii* (Jones et al., 2010). *Acetobacterium* spp., Bacteroidales, Firmicutes, *Methanolobus*, and *Methanosarcina* spp. were found in the coal seam water of Cook Bay, Alaska, United States (Dawson et al., 2012). Fuertez et al. (2018) found that *Methanofollis* and *Methanobacterium* were dominant in the optimized methane-producing bacteria solution. Although the microbial structure of CBM is extensively studied, the relationship between the increase in methane production and microbial flora, especially the reason why ethanol stimulates the increase in methane yield, is seldom reported.

This paper describes a process for biogas generation from coal. Small amounts of ethanol were added to a medium transforming coal to methane. This study aims to investigate the following: (1) the effect of adding ethanol on methane production, (2) the changes in the physical properties of coal during bioconversion, and (3) the bacterial and archaeon community changes before and after adding ethanol and the changes of gas production pathways.

## Materials and Methods

### Coal and Microflora Samples

The lignite samples were collected from the Shengli coal mine located in Xilinhot City, Inner Mongolia. The coal briquettes were crushed and sieved to prepare coal powder with a particle size of 180–250 μm (60–80 mesh), vacuum-dried at 80°C for 24 h, and placed in a desiccator for use.

The coal formation water was collected from CBM production wells in the Sihe mine (Jincheng, China) of Qinshui Basin. Enrichment experiments in triplicate were performed for approximately 3 months. The enrichment culture was used as the inoculation source of this study. The specific method is the same as that of Yang et al. (2018).

### Experimental Setup and Operation

Twelve microcosms (500 mL bottle) were established. Each microcosm contained 20 g of coal, 250 mL of the medium and 50 mL of inoculum. The 12 bottles were divided into four groups, and three parallels in each group. The first, second, third and the fourth group, were added with ethanol at 0 (0%, V/V), 1.5 (0.5%, V/V), 3.0 (1.0%, V/V), and 6.0 mL (2.0%, V/V), respectively, and named them group A, group B, group C, and group D in turn.

In addition, a blank control group was created in which no coal was added, but added with ethanol at 3.0 mL (0%, V/V) and the other conditions were the same, and named it group coal-free, and three parallels in this group. All bottles were purged with nitrogen completely and then incubated at 30°C under static conditions. The medium contained the following (in g/L): yeast extract, 2.0 g; KH_2_PO_4_, 1.5 g; K_2_HPO_4_, 2.9 g; MgCl_2_, 0.4 g; NH_4_Cl, 1.8 g; cysteine, 3.0 g; resazurin, (0.2%) 2 mL; and trace element solution, 10 mL, pH = 7.0. The trace element solution formula is as follows: Nitrilotriacetic acid, 1.5 g; CaCl_2,_ 0.1 g; MgSO_4_⋅7H_2_O, 3.0 g; H_3_BO_3,_ 0.05 g; FeSO_4_, 0.1 g; NaCl, 1.0 g; COCl_2_, 0.1 g; MnSO_4_, 0.5 g; ZnSO_4_, 0.1 g; NaMO_4_, 0.05 g; AlK(SO_4_)_2_, 0.01 g; NiCl_2_, 0.1 g; and CuSO_4_, 0.01 g. The samples were continuously cultured for approximately 3 months, and from the 28th day, samples were prepared for DNA extraction. On days 7, 14, 20, 28, 35, 42, 49, 60, 86, and 92, the concentration of CH_4_ in the headspace was measured by gas chromatography (GC) in microcosms.

### Gas Composition Determination

Gas composition and content during gas fermentation were analyzed by the American Agilent GC-7890 gas chromatograph. Chromatographic parameters included the following: Agilent-Carbon PLOT column (60 m × 320 μm × 0.25 μm); the chromatographic inlet temperature was 150°C; the septum purge flow was 3 mL/min; the column oven temperature was 25°C, which was maintained for 7.5 min; the detector TCD temperature was 200°C, the injection volume was 0.5 mL, and the carrier gas was high-purity nitrogen.

### Ultimate, Proximate, and FTIR Analyses of the Coal Samples

Coal powder with a particle size of 180–250 μm was used for ultimate, proximate, and FTIR analyses after freeze drying. Ultimate and proximate analyses of the coal samples on a dry basis were completed by the Shanxi Institute of Coal Chemistry, Chinese Academy of Sciences. The proximate analysis was performed according to GB/T212-2001 (National Standards of China, 2001), whereas the ultimate analysis was performed according to GB/T476-2001 (National Standards of China, 2001).

An IR Prestige-21 IR Analyzer (Shimadzu, Japan) was used to monitor the alterations in chemical bonds in the coal, with the KBr pellet method used in the mid IR region (4000–400/cm). KBr pellets were made from 0.0250 g of coal samples and 2.000 g of KBr after accurate weighing and were mixed and powdered in an agate mortar at 80 kN. Interfering background bands in KBr were quantified with a pure KBr pellet and subsequently subtracted from the spectra of samples by using the Shimadzu IR solution software. Each spectrum resulted from the average of 10 scans recorded in the 4000–400/cm spectral range with a resolution of 4/cm. The measurement mode was an interferogram.

### DNA Extraction

The changes in the microbial community structure during coal bioconversion in different ethanol contents were analyzed. Microorganisms were sampled at the 0 day and 28th, 35th, 42nd, 49th, 60th, 86th, and 92nd day (named as 0, 28, 35, 42, 49, 60, 86, and 92, respectively) of processing in microcosms of group A, group B and group C. Total DNA was extracted from samples by using the Power Soil DNA Isolation Kit (MO BIO Laboratories, Carlsbad, CA, United States) according to the manufacturer’s protocol. DNA quality and quantity were assessed by the ratios of 260 nm/280 nm and 260 nm/230 nm, respectively. The extracted genomic DNA was detected in 0.7% agarose gel to ensure size and integrity. DNA was stored at −80°C until further use.

### Amplification of 16S rRNA Genes

The extracted genomic DNA was used as a template for the PCR amplification of bacterial and archaeal 16S rRNA genes. The V3–V4 variable regions of the bacterial 16S rRNA genes were amplified by the primer pair forward primer 338F (5′-ACTCCTACGGGAGGCAGCA-3′) and reverse primer 806R (5′-GGACTACHVGGGTWTCTAAT-3′). The sequence of the archaeal V3-V4 region was amplified using the forward primer Arch349F (5′-GYGCASCAGKCGMGAAW-3′) and the reverse primer Arch806R (5′-GGACTACVSGGGTATCTAAT-3′). PCR amplification was performed in a total volume of 50 μL, which contained 10 μL of Buffer, 0.2 μL of Q5 high-fidelity DNA polymerase, 10 μL of high GC enhancer, 1 μL of dNTP, 10 μM of each primer, and 60 ng of genome DNA. Thermal cycling conditions were as follows: an initial denaturation at 95°C for 5 min, followed by 15 cycles at 95°C for 1 min, 50°C for 1 min, and 72°C for 1 min, with a final extension at 72°C for 7 min. The PCR products from the first-step PCR were purified through VAHTSTM DNA clean beads. A second-round PCR was then performed in a 40 μL reaction that contained 20 μL of 2 × Phusion HF Master Mix, 8 μL of ddH_2_O, 10 μM of each primer, and 10 μL of PCR products from the first step. Thermal cycling conditions were as follows: an initial denaturation at 98°C for 30 s, followed by 10 cycles at 98°C for 10 s, 65°C for 30 s, and 72°C for 30 s, with a final extension at 72°C for 5 min. Finally, all PCR products were quantified by Quant-iT^TM^ dsDNA HS reagent and then pooled together. The amplification systems and methods of bacteria and archaea were the same, except for the different primers. The qualities of the amplified PCR products were checked through electrophoresis in 1% agarose gel. High-throughput sequencing analysis of genes was performed on the purified, pooled PCR products using the Illumina Hiseq 2500 platform (2 × 250 paired ends) at Biomarker Technologies Corporation, Beijing, China.

### High-Throughput Sequencing and Analysis

After sequencing, FLASH v1.2.7 software was used to splice the reads of each sample, followed by Trimmomatic v0.33 software to filter the spliced raw tags to obtain high-quality tag data, and finally, UCHIME v4.2 Software to identify and remove chimeric sequences to obtain the final valid high-quality sequence. Further, UCLUST in QIIME (v. 1.8.0) software was used to cluster high-quality sequences at 97% similarity level, obtain OTU, and perform species annotation and abundance analysis. On the basis of the OTU number results, the bio-α diversity (including Chao1 value, ACE value, Shannon index, and Simpson index) and β-diversity of the sample were evaluated.

### Acquisition of Serial Number

The Illumina sequencing data were submitted to the Sequence Read Archive (SRA) of the National Center for Biotechnology Information (NCBI). The accession number is SRS3948544.

## Results

### Effects of Different Concentration of Ethanol on Methane Production

**FIGURE 1:**
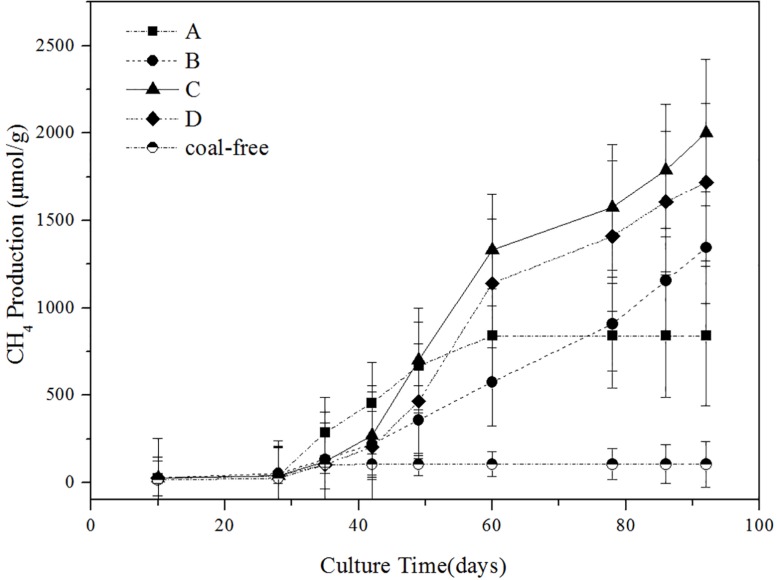
Influence of different ethanol contents on methane production. A: no added ethanol, B: with added 0.5% ethanol, C: with added 1% ethanol, D: with added 2% ethanol, coal-free: no added coal. Error bars indicate the standard deviation of three parallel samples.

The impact of different concentration of ethanol on the methane production is shown in Figure 1. As illustrated, the gas production process can be roughly divided into three stages: the first stage (0–40 days) of slow growth, the second stage (40–60 days) of rapid growth, and the third stage (60–90 days) of restrained growth. This observation is consistent with the conclusion drawn by Fuertez et al. (2018). In the first stage, the microflora needed to adapt to the new environment and their number was fewer, so less gas was produced (Take group C as an example, the cumulative methane production was 4.2 mg/g). In the second stage, gas production reached its peak (Take group C as an example, the cumulative methane production was 20.9 mg/g). The gas production of the experimental groups with added ethanol was significantly higher than that of the group A. In particular, the gas production of the group C was as high as 1332.06 μmol/g. Thus, adding ethanol obviously promoted the generation of biogas. On the third stage, the gas production tended to increase slowly, possibly because many inhibitors were produced during the fermentation, thereby restricting the formation of methane (Ma et al., 2015). On the 92nd day of gas-producing fermentation, the cumulative methane production of ethanol content at 0, 0.5, 1, and 2% was 839.68, 1344.56, 2002.55, and 1718.64 μmol/g, respectively. So in terms of gas production, adding 1% ethanol was the best. The coal-free control group produced almost no methane, so it can confirmed that the methane produced in the experimental group comes from coal rather than added compounds in the microcosms.

### Proximate and Elemental Analysis

The coal samples were characterized by approximate and elemental analysis, as shown in Table 1. Raw coal samples contained approximately 13.22% water, 35.90% ash, 26.76% volatile substances, and 24.12% fixed carbon. Compared with raw coal samples, volatiles increased from 26.76 to 31.20%, even reached 33.75% in group C samples after gas fermentation, and the ash content fell from 35.90 to 25.14%. We speculated that the microbial community utilized coal as a substrate to generate some easily decomposed and oxidized organic matter, such as organic acids, aromatic hydrocarbons, alcohols, and carbohydrates. After the gas fermentation, the fixed carbon content increased to 28.31%, suggesting that microorganisms metabolized with coal as a carbon source and produced organic matter, which was then attached to coal particles. Oxygen content increased to 14.26%. Oxygen was the most abundant heteroatom, and the oxygen atoms in coal were connected by bonds such as ether, ester, carbonyl, and hydroxyl (Chen et al., 2017). The hydrogen, nitrogen, and sulfur contents did not remarkably change. In general, the proximate analysis and the element composition of coal before and after fermentation were significantly different, whereas the difference between the group C samples and the group A samples was obscure. Thus, the change of physical and chemical properties of coal was mainly affected by anaerobic fermentation.

**TABLE 1:** Proximate and elemental composition of coal samples.

**Analysis of proximate and elemental**	**Coal (lignite)**		
	**Raw coal**	**Group A**	**Group C**
Moisture%	13.22	15.35	14.66
Ash%	35.90	25.14	25.42
Volatile matter%	26.76	31.20	33.75
Fixed carbon%	24.12	28.31	26.17
Carbon%	35.81	40.05	40.23
Hydrogen%	2.73	2.82	2.83
Nitrogen%	0.50	0.88	0.80
Sulfur%	1.29	1.50	1.36
Oxygen%	10.55	14.26	14.70

### FTIR Characterization of the Coal Samples

**FIGURE 2:**
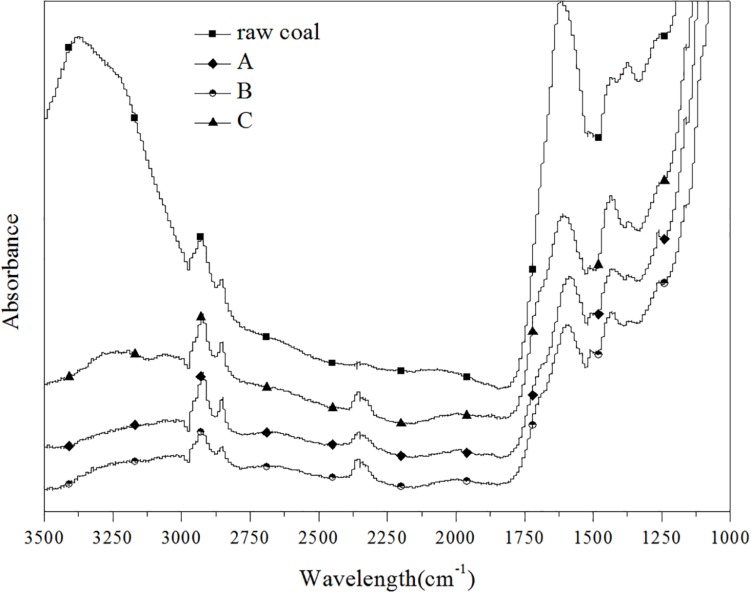
FTIR spectra in the range of 3,500–1,000/cm for lignite coal sample (raw coal, A, B, C).

Samples of raw coal, control (group A) and experimental groups (added ethanol-fermented samples) were selected for FTIR spectrum characterization, as shown in Figure 2. After anaerobic fermentation, compared with raw coal, the structure of coal in the control (without ethanol) and experimental group changed significantly. However, there was no structural difference between the control group and the experimental group. The peak heights and peak areas of different coal samples were different, but the approximate peak shapes were the same, indicating that they contained similar functional groups. The structural composition of lignite can be described by alkyl-C, aromatic-C, carbonyl-C, and O-alkyl-C contents (Haq et al., 2018). The characteristic peaks in the IR spectrum were mainly divided into three categories: (1) Aromatic hydrocarbon: The peak at 2,919/cm was related to the stretching vibration of aromatic C-H bond; 1,600/cm was the characteristic absorption peak of the aromatic ring. (2) Aliphatic hydrocarbons: The peaks at 2,856 and 1,440/cm were respectively related to the symmetrical and asymmetric bending vibration of the alkane C-H bond. (3) Oxygen-containing functional group: The peak at 3,375/cm represented the stretching vibration of the O-H bond of fatty alcohol and phenol, and 1,230/cm represented the stretching vibration of phenol and ether C-O bond. As generally known, -O-CH_3_ is the basic structural unit of coal (Strąpoć et al., 2011; Colosimo et al., 2016). After the biogas fermentation, the absorbance at all peaks decreased, indicating that the coal was consumed by the action of microorganisms, and the substances in the coal were utilized by the microorganisms. Two special peaks, that is, 3,375 and 2,300/cm, were considered. The peak at 3,375/cm clearly disappeared, suggesting that microorganisms can utilize aromatic hydrocarbons and long-chain fatty alcohols. A new peak appeared at 2,300/cm, which represented the asymmetric stretching vibration of the triple bond and the cumulative double bond, indicating that new small molecules, possibly an alkyne or an olefin, were produced during the gas fermentation process. The results suggested that the change in the structure of coal was mainly affected by anaerobic fermentation rather than by adding ethanol.

### High-Throughput Sequencing Analysis

High-throughput sequencing results showed that 2,522,076 high-quality sequences of 40 bacteria samples and 2,094,651 high-quality sequences of 40 archaea samples were obtained. The bacterial flora OTU was counted as 283, and the archaea flora OTU was counted as 22. All reads were deposited in the SRA of NCBI with the accession number SRS3948544.

### Characterization of Microbial Community Structure

**FIGURE 3:**
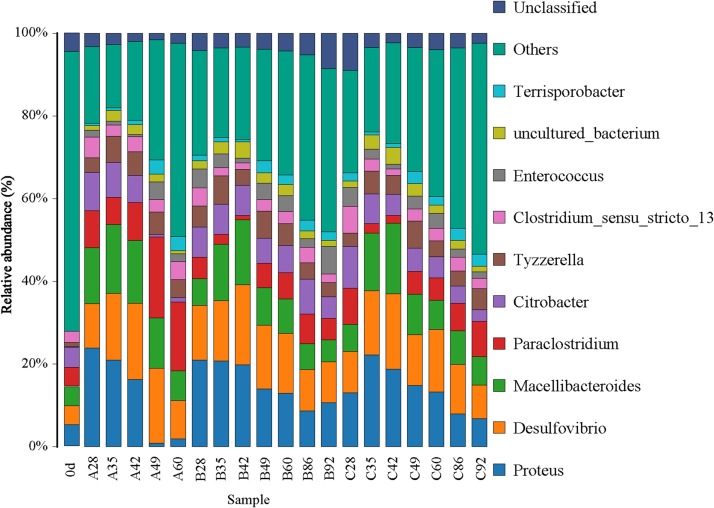
Bar chart of the relative abundance of the top 10 bacteria of each group in genus level.

**FIGURE 4:**
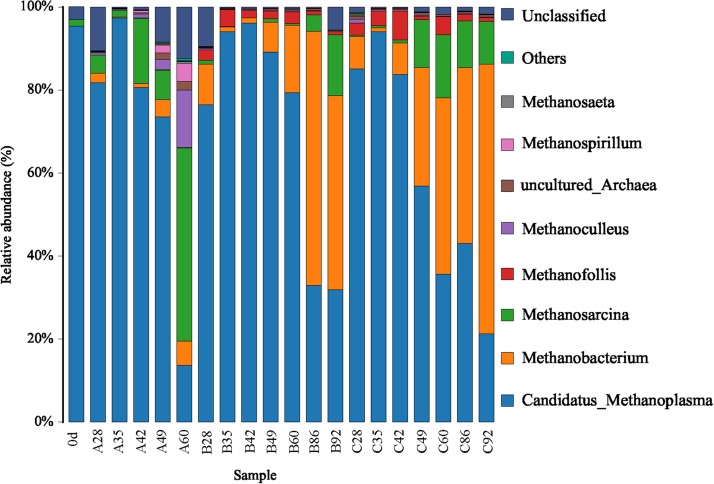
Bar chart of the relative abundance of the top 10 archaea of each group in genus level.

For convenient data analysis, we presented the sequencing results after the randomly selected samples. The bacterial microbial community was mainly composed of Firmicutes, Proteobacteria, and Bacteroidetes at the phylum level during the gas-producing fermentation process (Supplementary Figure S1). As shown in Figure 3, from the community abundance analysis of bacterial genus, the main bacteria included *Proteus*, *Desulfovibrio*, *Macellibacteroides*, *Paraclostridium*, *Citrobacter*, *Enterococcus*, and *Tyzzerella*. With the extension of fermentation time, the changes of species trend in the experimental groups A, B, and C were similar. It is suggested that ethanol had little effect on the structure of bacterial flora. Figure 4 shows that the archaea flora is mainly composed of *Candidatus-*Methanoplasma, *Methanobacterium*, *Methanosarcina*, and a small amount of *Methanofollis*. In the early stage of fermentation, *Methanosarcina* occupied a certain proportion in the control group, but it could hardly found in the experimental group. With the extension of fermentation time, the difference of the flora between the control group and the experimental group was obvious. *Methanosarcina* was a continuously increasing microorganism in the control group, however, it was *Methanobacterium* in the experimental group.

*Candidatus*-Methanoplasma dominated the advantage in the sample A28, gaining the percentage of 81.75%. As the fermentation time was prolonged, its percentage gradually decreased. After 60 days of fermentation, its percentage decreased to 13.66% (A60). Its content was decreasing with the prolonged fermentation time in groups A, B, and C. In group A, the percentage of *Methanosarcina* in A28 was 4.33%, that of *Methanosarcina* in A49 was 7.16%, and that of *Methanosarcina* in A60 was 46.55%. When the fermentation time was prolonged, *Methanosarcina* gradually became dominant. In groups B and C, *Methanobacterium* gradually became the dominant bacteria, with 46.80% in B92, and 64.92% in C92. Clearly, compared with group A, the pathway of methane formation in groups B and C was changed, which was caused by the addition of ethanol.

### Effect of Ethanol on Microbial Flora

**FIGURE 5:**
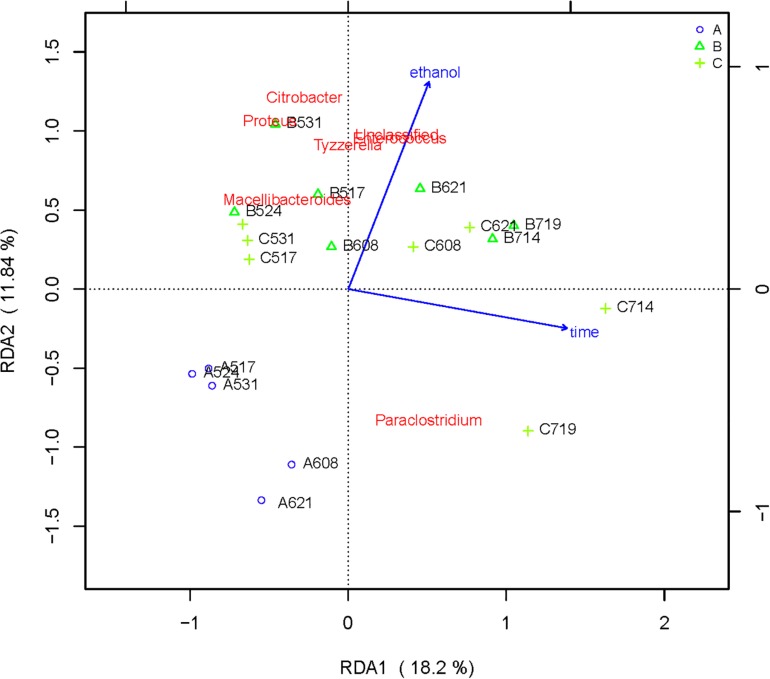
Redundancy analysis shows the relationships between environmental variables and each sample at the bacterial flora level, Label: A no added ethanol, B with added 0.5% ethanol, C with added 1% ethanol. Red words represent microbial species, blue words represent environmental factors (ethanol and time).

**FIGURE 6:**
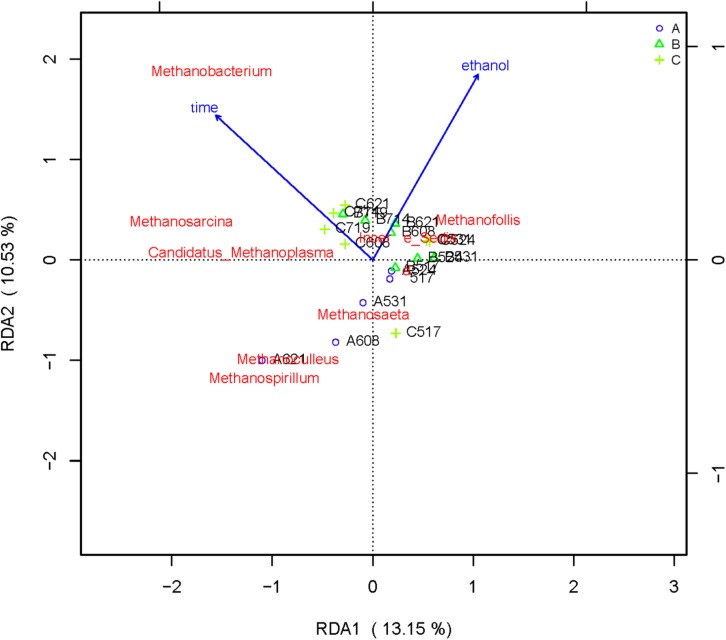
Redundancy analysis shows the relationships between environmental variables and each sample at the archaea flora level, Label: A no added ethanol, B with added 0.5% ethanol, C with added 1% ethanol. Red words represent microbial species, blue words represent environmental factors (ethanol and time).

RDA/CCA is a sorting method based on correspondence analysis and is mainly used to reflect the relationship between flora or sample and environmental factors. RDA/CCA analysis and mapping use the R language vegan package. The results of RDA/CCA analysis of species diversity between samples at the genus level are as follows. The RDA/CCA diagram of bacteria is shown in Figure 5, and the RDA/CCA diagram of archaea is shown in Figure 6. The relationship between points and points in the figure is represented by distance, and the closer the distance was, the more similar the sample was. The relationship between ray and another ray is represented by an included angle, obtuse angle represents negative correlation, and acute angle represents positive correlation.

The angle between the two environmental factors in the two graphs is an acute angle, indicating the positive correlation of the two environmental factors, namely, the addition of ethanol and the fermentation time. In the two figures, the distance between group B and group C is short, and the distribution is concentrated. Therefore, the sample composition of the added ethanol is similar according to bacterial and archaeal level analyses. As depicted in Figure 5, all of the bacterial microbial strains including *Citrobacter*, *Proteus*, *Desulfovibrio*, *Macellibacteroides*, *Enterococcus*, *Clostridium*, and *Tyzzerella*, and the environmental factor ethanol nearly presents an acute angle of rays, indicating a positive correlation between these bacteria and ethanol. Combined with the species distribution histogram of bacteria (Figure 3), the species composition of groups A, B, and C did not notably change, indicating that although the microbial flora positively correlated with ethanol, the effect was obscure.

From the RDA diagram of archaea (Figure 6), *Methanosarcina* was positively correlated with the fermentation time but negatively correlated with ethanol. This result is consistent with those shown in the distribution histogram of archaea (Figure 4). The *Methanosarcina* increased with the extension of fermentation time in group A, but not in groups B and C. A positive correlation existed between *Methanobacterium* and fermentation time, as well as ethanol. Combined with Figure 4, the effect of ethanol on *Methanobacterium* was far more powerful than that of the fermentation time.

### Correlation Analysis Between Gas Production and Microbial Flora

**FIGURE 7:**
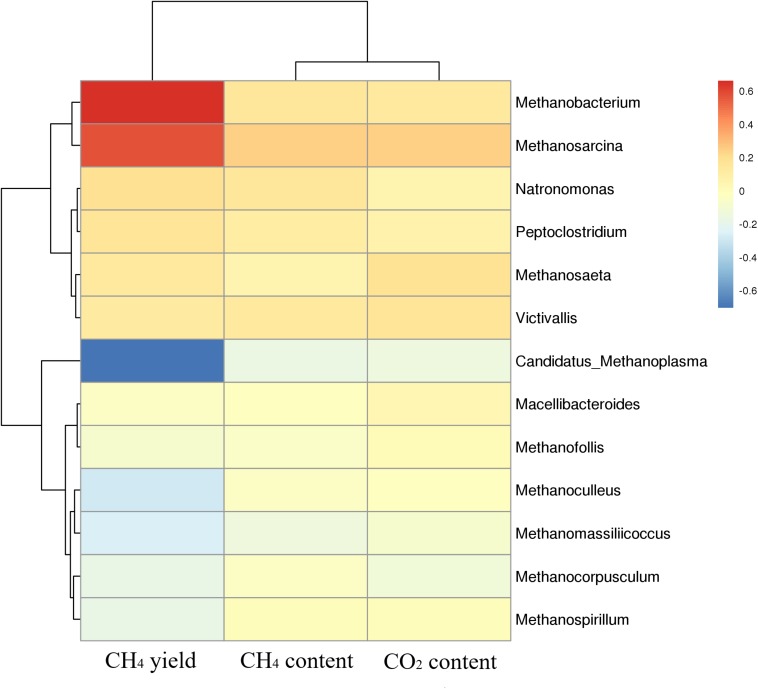
Cluster analysis of methanogenic flora with CH_4_ yield, CH_4_ content, and CO_2_ content. Red color represents slight difference among methanogenic flora, and blue color represents apparent differences.

**FIGURE 8:**
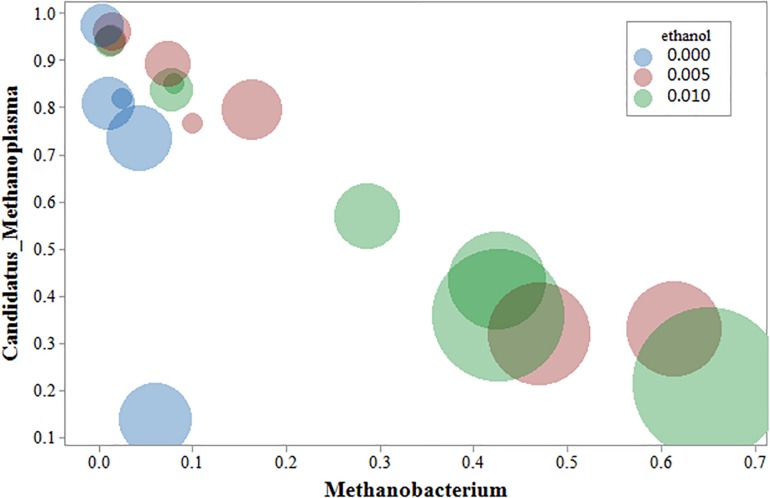
Bubble chart of three variables: methanogens, ethanol content, and methane yield. The abscissa represents *Methanobacterium*; the ordinate represents *Candidatus*-Methanoplasma, the bubble size represents the amount of methane yield, blue represents the Group A sample, red represents the B sample, and green represents the C sample.

Cluster analysis of methanogenic flora with CH_4_ yield, CH_4_ content, and CO_2_ content was performed. As shown in the Figure 7, two dominant types of microbial communities were clearly noted in the process of gas production, namely, *Methanobacterium* and *Methanosarcina*, which negatively correlated with *Candidatus*-Methanoplasma. Although the distribution histogram of archaea showed that *Candidatus*-Methanoplasma accounted for a large proportion of all samples (Figure 4), its presence did not help the production of methane gas. According to the above analysis, the addition of ethanol led to the differences in the structure of methanogenic community in the samples, and the *Methanobacterium* increased significantly. To further study *Candidatus-*Methanoplasma and *Methanobacterium* playing the role in the process of gas production, we made an intuitive analysis of the two bacterial populations, as shown in Figure 8. It showed the relationship between methane yield, ethanol content, and methanogenic community in all samples. The difference in bubble size was large in the figure, indicating that a large difference existed in methane production among the samples; the larger bubbles were clustered in the high position of *Methanobacterium* and in the low position of *Candidatus*-Methanoplasma, and their color belonged to the category of 1% ethanol content. Without the ethanol-added sample, bubbles were concentrated in the high position of *Candidatus*-Methanoplasma and in the low position of *Methanobacterium*, and they had smaller bubbles. With 0.5% ethanol-added sample, bubbles are gradually larger as the *methanobacterium* gradually increases. Results further indicated that the presence of *Candidatus*-Methanoplasma might not be conducive to the production of methane. Additionally, the presence of *Methanobacterium* was beneficial to the increase of gas production, and its positive effect was far greater than that of the negative effect of *Candidatus*-Methanoplasma.

## Discussion

Microbial CBM production technology can produce new CBM and effectively alleviate energy stress. Scholars have performed many experimental studies on increasing CBM yield by optimizing gas production conditions from various aspects, such as culture temperature, pH of nutrient solution, coal granularity, solid–liquid ratio, and addition of different microelements, to achieve increased microbial CBM production (Ünal et al., 2012; Ghosh et al., 2014). As a kind of green, environment-friendly and cheap organic matter, ethanol is also used in CBM production. Some scholars stimulate microbial activity by adding ethanol, but other organic matters, such as methanol and isopropyl alcohol, are added at the same time (Bi et al., 2017). Such a case cannot be expressed qualitatively because of the increased production of CBM caused by the addition of ethanol, and possibly because of the interaction of ethanol with other substances. In this study, the composition of the medium was relatively single without adding other organics or surfactants, thereby allowing to better explore the role of ethanol. Most researchers are focused on the optimization of the conditions to increase CBM yield, but the changes in microbial flora structure during the optimization of gas production are poorly studied. In this current study, the changes of microbial flora were tracked in real time during the gas fermentation process. We found that the addition of ethanol led to significant changes in the methanogenic archaeon flora, thereby changing the gas production pathway.

This study once again confirmed the effect of ethanol on CBM production, and on the basis of previous studies, the mechanism of ethanol to increase methane production was more deeply studied. The results of biogenic gas experiment indicated that methane production in the experimental group was higher than that in the experimental group without ethanol. By comparison, the gas yield of 1% ethanol content was the highest, reaching up to 2002.55 μmol/g. The yield of methane was similar to that of Bi et al. (2017), and the yield increase effect was also favorable. It was worth mentioning that methane production of the control was higher than that of the experiment groups B and C between days 30 and 50, it might be due to the microbial flora required a longer time to adapt to the environment with ethanol, which led to the phenomenon of the lag in producing methane in the experimental group in the early fermentation. In addition, from the change of H_2_ content (Supplementary Table S1), it was hardly detected in the control group, while the presence of H_2_ could be detected in the experimental group before 50 d. It is suggested that ethanol stimulated the production of H_2_ in the initial stage. Since the 50th day *Methanobacterium* began to increase in the experimental group, which is a typical hydrotropic archaea that can use H_2_ to produce methane. Due to the increase of quantity of *Methanobacterium*, it utilized H_2_ accumulated in the earlier stage to produce more methane. Simultaneously, we also tracked the change in the number of flora in the whole gas generation stage through QPCR (Supplementary Table S2). The quantity of microflora increased with the extension of fermentation time. The quantity of flora in the experimental group with ethanol was more than that of control under the same fermentation time. It can be speculated that ethanol plays a role in stimulating microbial flora growth throughout the fermentation process. Although this study contained yeast extract and ethanol, it might provided a carbon source for bacterial growth. In the study of Zhang et al. (2019), it has been confirmed that the majority of methane was from the coal itself rather than the MS medium and added ethanol. The amount of ethanol added in the whole experiment had a limit, not the more the better. In Zhang et al. (2016a) experiment, he found that the best effect was achieved when the amount of ethanol was 100 mM, but the methane production was inhibited when the amount of ethanol was 300 mM.

In the analysis of physical and chemical properties of coal, the structure of coal changed greatly after fermentation, but it had no effect on the structure of coal after adding ethanol. In addition to stimulating microorganisms, ethanol played an important role as an organic solvent, which dissolved small biodegradable molecules in the coal matrix and enhanced their bioavailability (Takanohashi et al., 2000). It was only a physical change and would not lead to the changes in the coal structure. In this study, the microcosms contained 20 g coal, after fermentation for 90 daya, the average cumulated methane production of the experimental group with 1% ethanol was 897.2 mL, while that of the control group was 376.2 mL. Compared with the control group, although the methane production was more than double, the increased carbon content of methane only accounted for 1.86% of the total coal. It was conceivable that its impact on the overall structure of coal was negligible (Supplementary Figure S2).

According to the analysis results of bacteria, the participating bacteria mainly included Firmicutes, Proteobacteria, Bacteroidetes, and a small amount of Synergistetes in the whole process of gas production. They are very common in the study and cultivation of CBM (Fuertez et al., 2018). The Firmicutes microorganisms mainly participated in the generation of some mixed acids, alcohols, and neutral substances, it plays an important role in the degradation of coal and contributes to the degradation of coal into aromatic compounds, aliphatic compounds and alkanes (Colosimo et al., 2016). Proteobacteria are a kind of abundant bacteria. In general, Proteobacteria in CBM are mainly of β-, γ- and δ- mycetozoa for syntrophism type (Colosimo et al., 2016). The ability of the bacteria to degrade is variable, they can degrade benzene, aromatic, alcohols and other compounds, and use nitrate as the electron acceptor. The decrease of the peak value at 3,375/cm in the infrared spectrogram was probably related to the effect of Proteobacteria. The bacteria belonging to this phylum in the mixed flora of samples included *Paraclostridium*, *Tyzzerella*, and *Enterococcus*. Proteobacteria is a kind of bacteria with abundant species. In general, the deformation bacteria in biogenic CBM are mainly composed of syntrophic deformation bacteria such as β- and γ-δ- deformation bacteria. Bacteroidetes, which is common in sediments, is a large class of chemoautotrophic microorganisms. The bacteria involved in this phylum in all samples were *Macellibacteroides* and *Citrobacter*.

From the taxonomic level of the bacteria genus, the participating bacteria mainly included *Proteus*, *Desulfovibrio*, *Macellibacteroides*, *Paraclostridium* and *Citrobacter*, with a small amount of *Enterococcus* and *Tyzzerella*. As a strictly anaerobic bacterium, *Desulfovibrio* is a δ- mycetozoa and a sulfate-reducing bacterium that can only use sulfate for respiration (Colosimo et al., 2016). *Desulfovibrio* could also use acetic acid, H_2_, it is possible to generate some unsaturated alkane compounds, thereby promoting the degradation of macromolecular coal. Members of *Desulfovibrio* have been identified in Ishikari Basin-Japan (Vick et al., 2018). In general, δ-proteobacteria can be found in oil fields, coal tar waste waters, coal beds, and formation waters (Jones et al., 2010). *Clostridium* is a kind of anaerobic bacterium that can produce spores with extensive catalytic and metabolic characteristics and degrades starch, chitin, xylose, and cellulose (Zhang et al., 2015). *Clostridium* BC1 and *Clostridium* scatologenes with heavy metal reduction and nitrogen fixation were found from coal (Küsel et al., 2000). *Clostridium* is dominant in stratigraphic water in Western Canada (Penner et al., 2010).

According to the archeal analysis, the archaea were mainly composed of Euryarchaeota at the phylum level and of *Candidatus*-Methanoplasma, *Methanobacterium*, *Methanosarcin*a, and a small amount of *Methanofollis* at the genus level. *Candidatus*-Methanoplasma belongs to Methanomassiliicoccales, which is currently the seventh type of methanogen (Lang et al., 2014). This type of bacteria is composed of obligate hydrogen-dependent methylotrophic bacteria. From the nutritional point of view, the methanogens are mixed nutrient type (Noel et al., 2016). Although the inoculum used in this study was the same as that used by Yang (Yang et al., 2018), *Candidatus*-Methanoplasma was not found in his study. This kind of bacteria has never been found in the study of the microbial flora of CBM. This species exists in the stomach of ruminants and in human feces (Noel et al., 2016). Herein, this kind of bacteria was unfavorable to the formation of methane from coal. The inoculation source used in this study was an enriched and domesticated culture medium. Therefore, we speculated that the bacteria were produced during enrichment and domestication.

Adding ethanol greatly affects the microbial community of archaea. Group A differed greatly from groups B and C. *Methanosarcina* and *Methanobacterium* belong to two completely different archaea. *Methanosarcina* has a wide range of available substrates, in addition to reducing H_2_/CO_2_, decomposing methyl compounds, and also decomposing acetic acid (Park and Liang, 2016). In the study of coal mining, the degradation of acetic acid pathway is the main one because acetate is more readily available than hydrogen (Beckmann et al., 2011). This finding suggests that *Methanosarcina* more likely followed the acetoclastic methanogenesis. Therefore, we speculated that with the extension of fermentation time, the group A was dominated by the methane production pathway of acetic acid type. By contrast, *Methanobacterium* is a typical hydrotrophic methanogen (Kimura et al., 2010). *Methanobacterium* was also discovered in the production of biogas from abandoned coal piles. The advantage of *Methanobacterium* is that similar to Firmicutes, they were consumed by during hydrogen production (Zheng et al., 2016). Therefore, the ethanol-added experimental group was mainly concentrated on the hydrotrophic methane-producing pathways.

## Conclusion

Our study confirmed that the addition of ethanol to the coal-enriched culture could stimulate microorganisms to increase the production of coal-to-methane. Anaerobic fermentation had a great effect on the structure of coal, but the addition of ethanol mainly increased the bioavailability of coal and had little effect on the main structure of coal. The 16S rRNA gene sequencing data showed that ethanol had little effect on bacterial microflora, but changed the microflora structure of archaea significantly, changing the gas-producing pathway from acetoclastic to hydrogenotrophic. This study revealed the intrinsic mechanism of ethanol to increase CBM and provided assistance for future research. Ethanol is non-toxic, inexpensive, and can be used in large-scale operations either *in situ* or *ex situ*.

## Author Contributions

XY designed the experimental plan, analyzed the results and read the final manuscript. QL made the experimental trials and wrote the manuscript. YC supported the technical part. BW provided some biological materials, participated to the experimental plan and read the final manuscript.

## Conflict of Interest

BW was employed by company Yi’an Lanyan Coal and Coalbed Methane Simultaneous Extraction Technology Co., Ltd. The remaining authors declare that the research was conducted in the absence of any commercial or financial relationships that could be construed as a potential conflict of interest.

## Supplementary Material

FIGURE S1 Bar chart of the relative abundance of the top 10 bacteria of each group in phylum level.

FIGURE S2 Figure of increasing the carbon content of methane as a percentage of total carbon content.

TABLE S1 The content of H_2_.

TABLE S2 Quantitative results for bacteria and archaea (log x ± s).
