# Supplementary figures and images for: Alteration of Methanogenic Archaeon by Ethanol Contribute to the Enhancement of Biogenic Methane Production of Lignite
Source: Front Microbiol. 2019 Oct 10;10:2323. doi: 10.3389/fmicb.2019.02323 (PMC6796574; doi:10.3389/fmicb.2019.02323)

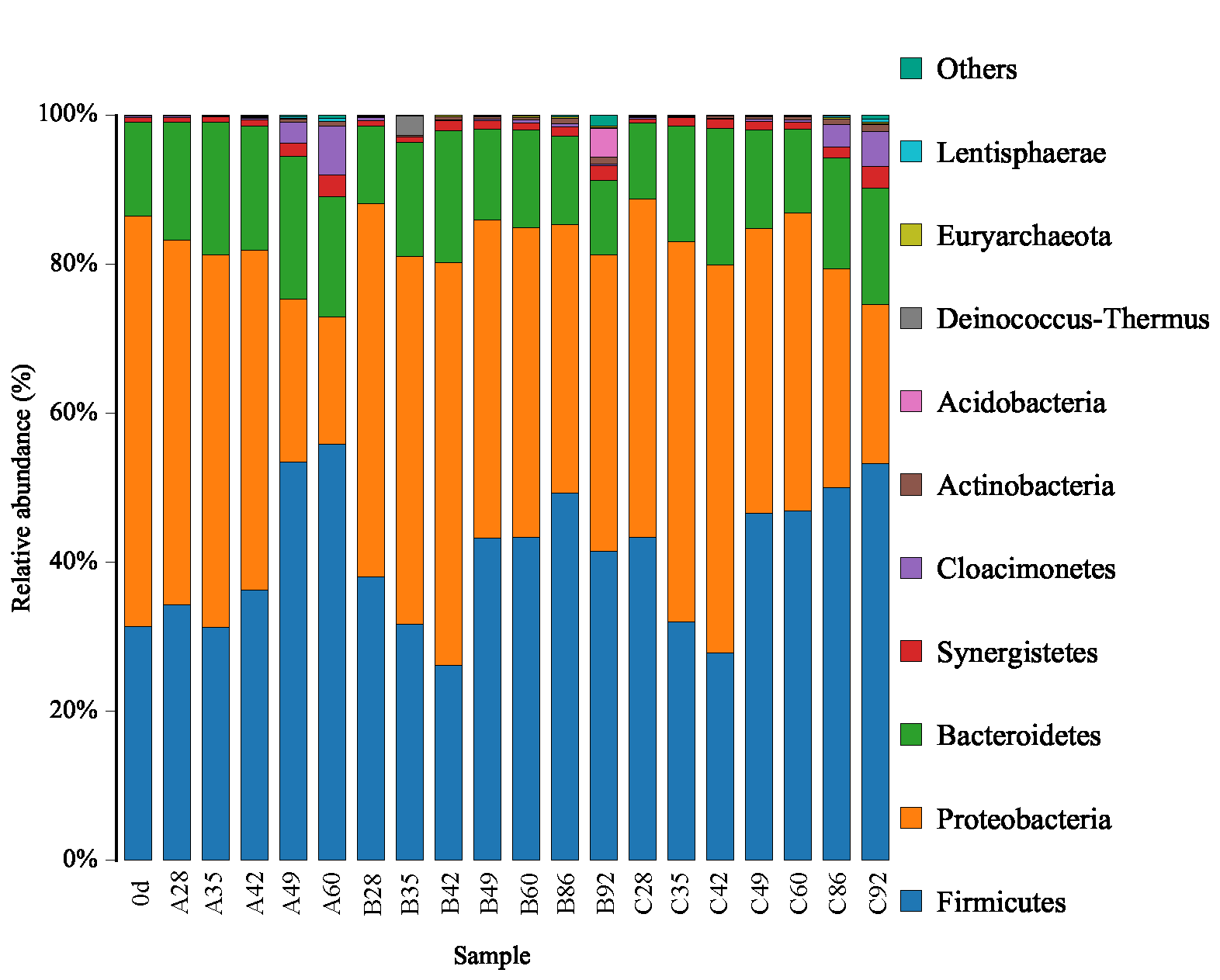

Supplement: FIGURE S1 — Bar chart of the relative abundance of the top 10 bacteria of each group in phylum level. [file Image_1.TIFF]

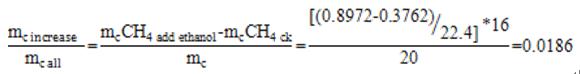

Supplement: FIGURE S2 — Figure of increasing the carbon content of methane as a percentage of total carbon content. [file Image_2.JPEG]
