# Supplementary material for: Alteration of Methanogenic Archaeon by Ethanol Contribute to the Enhancement of Biogenic Methane Production of Lignite
Source: Front Microbiol. 2019 Oct 10;10:2323. doi: 10.3389/fmicb.2019.02323 (PMC6796574; doi:10.3389/fmicb.2019.02323)
Supplement: TABLE S1 — The content of H2. [file Table_1.DOCX]

| Time  (days) | Ethanol content | | | | |
| --- | --- | --- | --- | --- | --- |
|  | 0 | 0.5 | 1 | 2 |  |
|  | H_2_/% | H_2_/% | H_2_/% | H_2_/% |  |
| 10 | 0.00 | 0.16 | 0.33 | 0.15 |  |
| 28 | 0.01 | 0.15 | 0.36 | 0.17 |  |
| 35 | 0.02 | 0.24 | 0.32 | 0.30 |  |
| 42 | 0.00 | 0.04 | 0.58 | 0.06 |  |
| 50 | 0.00 | 0.17 | 0.49 | 0.25 |  |
| 60 | 0.09 | 0.00 | 0.00 | 0.01 |  |
| 78 | 0.00 | 0.00 | 0.00 | 0.01 |  |
| 86 | 0.00 | 0.00 | 0.01 | 0.00 |  |
| 92 | 0.00 | 0.00 | 0.00 | 0.00 |  |

**Table 1 The content of H_2_**
