# Supplementary material for: Alteration of Methanogenic Archaeon by Ethanol Contribute to the Enhancement of Biogenic Methane Production of Lignite
Source: Front Microbiol. 2019 Oct 10;10:2323. doi: 10.3389/fmicb.2019.02323 (PMC6796574; doi:10.3389/fmicb.2019.02323)
Supplement: TABLE S2 — Quantitative results for bacteria and archaea (log x ± s). [file Table_2.DOCX]

**Table 2** **Quantitative results for bacteria and archaea (log x ± s)**

| Sample | Number of bacteria/mL | Number of archaea/mL |
| --- | --- | --- |
| A 28 | 3.02±0.78 | 2.22±0.39 |
| B 28 | 3.50±0.58 | 3.55±0.26 |
| C 28 | 4.71±0.35 | 3.78±0.42 |
| P value | 0.21 | 0.32 |
| A 35 | 3.46±0.40 | 2.55±0.31 |
| B 35 | 3.58±0.64 | 3.71±0.25 |
| C 35 | 3.72±0.22 | 3.73±0.32 |
| P value | 0.10 | 0.22 |
| A 42 | 3.85±0.57^a^ | 3.66±0.39^a^ |
| B 42 | 4.78±0.45^b^ | 3.73±0.56 ^b^ |
| C 42 | 5.67±0.27^b^ | 3.78±0.45 ^b^ |
| P value | 0.03 | 0.03 |
| A 49 | 3.28±0.65^a^ | 3.73±0.12^a^ |
| B 49 | 4.63±0.36 ^b^ | 3.94±0.47 ^b^ |
| C 49 | 4.79±0.32 ^b^ | 4.79 ±0.50 ^b^ |
| P value | 0.03 | 0.02 |
| A 60 | 3.63±0.60^a^ | 3.77±0.48^a^ |
| B 60 | 5.41±0.55 ^b^ | 4.88±0.36 ^b^ |
| C 60 | 5.87±0.39 ^b^ | 5.08±0.21 ^b^ |
| P value | 0.01 | 0.01 |
| Note: The same letter on the same column indicates that there is no significant difference (P>0.05), and the absence of the same letter indicates that there is the significant difference (P<0.05). The number in the sample name is the number of culture days. For example, A28 indicates that the group A sample is cultured for 28 days. | | |
